# Air pollution data: A dataset gathered through a crowd sensing platform

**DOI:** 10.1016/j.dib.2025.111683

**Published:** 2025-05-18

**Authors:** Slave Temkov, Pance Cavkovski, Petre Lameski, Eftim Zdravevski, Michael A. Herzog, Vladimir Trajkovik

**Affiliations:** a Ss Cyril and Methodius University in Skopje, Faculty of Computer Science and Engineering, North Macedonia; b Magdeburg Faculty of Computer Science, Magdeburg-Stendal University of Applied Sciences, 39011 Magdeburg, Germany

**Keywords:** Pollution, Air quality, Sensor networks, Crowd sensing, IoT platform

## Abstract

This paper introduces an extensive dataset on air pollution monitoring, collected through a crowd sensing IoT platform. The dataset contains real-time measurements of various pollutants, including PM_2.5_, PM_10_, NO_2_, O_3_, and CO, enriched with meteorological parameters such as temperature, humidity, and atmospheric pressure. Additionally, it includes noise level measurements, offering insights into urban noise pollution. The data, collected across multiple urban locations in Skopje, North Macedonia, spans from early 2018 to December 2024, providing both high spatial and temporal resolution. This dataset is a valuable resource for studying pollution trends, forecasting pollution levels, identifying pollution sources, and assessing the impact of urban planning on air quality. All in all, it supports research aimed at improving air quality and public health through data-driven decision-making and policy development.

Specifications Table

**Table:** 

Subject	Earth & Environmental Sciences
Specific subject area	*Air Pollution, Sensors*
Type of data	*Table*
Data collection	*Data acquired from the Pulse.eco* [1] *crowd sensing IoT platform using a network of air quality sensors*
Data source location	*Various urban areas in Skopje, North Macedonia*
Data accessibility	Repository name: Repository of UKIM Data identification number: 20.500.12188/32263 Direct URL to data: https://repository.ukim.mk/handle/20.500.12188/32263 Backup URL: https://github.com/temkovs/skopje-air-pollution-data

## 1 Value of the Data


 

• Air pollution is a growing global concern, with significant adverse effects on public health, including respiratory and cardiovascular diseases, and impacts on overall quality of life [2,3]. This dataset enables policymakers, researchers, and public health advocates to make evidence-based decisions that can lead to improved living conditions and reduced health risks through targeted interventions and policy measures.
• Traditional methods for air quality monitoring often rely on sparse and expensive monitoring stations, limiting spatial and temporal coverage [4]. The presented dataset, gathered using a dense network of cost- effective sensors, provides an alternative by offering high-resolution, real-time data that can complement or even replace existing monitoring frameworks. This data, also possibly integrated with the data from traditional sources, can help to uncover complex pollution patterns through multimodal analysis and predictive modelling [5].
• This dataset is particularly valuable for the development and validation of machine learning models aimed at forecasting pollution levels, detecting pollution sources, and assessing the impact of urban planning decisions on air quality [[5], [6], [7], [8]]. These models can serve as decision making tools for urban planners and environmental institutions to improve the sustainability of cities.
• Beyond its technical applications, the dataset supports public awareness campaigns by providing transparent, accessible, and useful information about air quality. This can drive community engagement and promote bringing everyone’s efforts together to address environmental challenges, with an accent to the importance of citizen participation in the fight against pollution [9].

## 2 Background

Air pollution poses a significant threat to public health and urban sustainability, necessitating accurate and continuous monitoring. Traditional air quality monitoring relies on sparse networks of governmental stations, which, due to their high costs and limited spatial coverage, cannot provide a comprehensive understanding of pollution dynamics. To address these limitations, Pulse.eco, a crowd-sensing IoT platform, was developed to enable large-scale, high-resolution monitoring of air pollution using a distributed network of low-cost sensors.

So now we want to share a big portion of the data that has been collected through this platform up until now, so that also people that do not have the technical skills to access it through the APIs can get it. But also, we want to provide description and interpretation of the data that we are gathering.

## 3 Data Description

The air pollution dataset consists of real-time measurements collected from a dense network of IoT sensors deployed across multiple urban locations in Skopje, North Macedonia. This dataset serves as a valuable re- source for studying air quality trends in a region facing significant pollution challenges due to urbanization, industrial activities, and traffic congestion. It provides comprehensive air quality information, focusing on key pollutants known to pose severe risks to public health, such as particulate matter (PM_2.5_ and PM_10_), nitrogen dioxide (NO_2_), ozone (O_3_), and carbon monoxide (CO). Additionally, the dataset includes meteorological parameters like temperature, humidity, and atmospheric pressure, which can be used to understand the complex interactions between climate conditions and pollution levels. Furthermore, it also captures noise levels, offering insights into urban noise pollution, another ambient factor with significant implications for public health and well-being.

Each sensor in the network is configured to record data at a certain interval - every 15 min, ensuring high temporal granularity, which enables the identification of pollution peaks during rush hours or specific weather events, providing valuable insights into the time dynamics of the pollution.

The dataset covers a significant time frame - the period from the beginning of 2018 to December 2024. This extensive temporal coverage allows for long-term trend analysis and seasonal pattern detection, enabling researchers and policymakers to assess the effectiveness of air quality improvement measures over time. By combining the granularity of the measurements with the breadth of coverage, this dataset is poised to support a wide range of applications, including pollution forecasting, health impact assessments, and urban planning strategies aimed at mitigating air quality issues.

The different possible types of values (metrics) of the data points are as follows:

• PM_2.5_ or PM_10_: Measured in µg/m^3^, representing fine particulate matter concentrations at 2.5 microns and 10 microns, respectively. (value types: ‘pm25’ and ‘pm10’).
• NO_2_: Measured in parts per billion (ppb), an important marker for traffic-related pollution. (value type: ‘no2’).
• O_3_: Ozone, measured in ppb, giving insight into the oxidative capacity of the urban air. (value type: ‘o3’).
• CO: Carbon monoxide, measured in ppm, indicating the presence of combustion-related pollutants. (value type: ‘co’).
• Temperature: Measured in degrees Celsius (°C), providing essential context for understanding the impact of temperature on pollutant dispersion and chemical reactions in the atmosphere. (value type: ‘temperature’).
• Atmospheric Pressure: Measured in hectopascals (hPa), offering additional context for meteorological conditions that affect air quality dynamics. (value type: ‘pressure’).
• Noise: For this there are 2 different entries for each measurement, with 2 different value types - ‘noise’ and ‘noise_dba’. The ‘noise’ value is the raw recorded value by the sound sensor, which represent just the noise “energy” that was captured. With some standard calculations and assumptions, the ‘noise_dba’ is generated as a measure for the noise in decibels (dB). This is done using the following equation: $\text{noise}\_\text{dba}=\text{round}(37.08\cdot \log_{10}\left(\text{noise}\right)-14.7)$. The equation is developed by comparing the measures from the sensors against those of an external dBA sound level meter, where the source of noise were different levels of constant traffic noise. The preciseness of this equation would depend on many different factors. However, here wejust want to measure an indicative level of perceptive noise. These measures are capturing the urban noise pollution levels, which can impact human health and quality of life.

The dataset is organized into two CSV files, each serving a distinct purpose.

The first file contains the core measurement data, which records pollutant or atmospheric parameter readings collected from individual sensors at specific time points. Each row in this file represents a single measurement and includes the following four columns (in this specific order):

• SensorId - a unique identifier (string) of the sensor where the measurement came from
• Type - the type of the measurement (pm10, pm25, co, humidity and so on).
• Value - the value that was measured.
• Stamp - the timestamp (UTC) of when exactly was the measurement recorded.

Each row in this file contains only one specific measurement. To retrieve all measurements recorded by a specific sensor node at a particular time, the data must be matched using the **SensorId** and **Stamp** columns.

For example, if we see the following row in the ‘data.csv’ file

200cdb67-8dc5-4dcf-ac62-748db636e04e,pm10,10,2018-05-14T07:03:27+02:00

It means that the sensor with id “200cdb67-8dc5-4dcf-ac62-748db636e04e” measured a value of 10 for PM_10_ at the exact time 2018-05-14T07:03:27+02:00.

The second file contains metadata associated with the sensors, provid- ing additional context about their characteristics and deployment. This file consists of the following six columns (in this specific order):

• SensorId - a unique identifier (string) of the sensor.
• Type - the type ID of the sensor.
• Position - geographical coordinates of where the sensor is placed (latitude, longitude).
• Description - short description or name of the sensor.
• Comments - additional notes or remarks about the sensor.
• Status - the operational status of the sensor.

The Type of a sensor indicates its classification based on the platform or technology used for data collection. It can have one of the following values:

• “0” - MOEPP measurement station.
• “1” - SkopjePulse LoRaWAN based sensor, version 1.
• “2” - SkopjePulse WiFi based sensor, version 1.
• “3” - pulse.eco WiFi based sensor, version 2.
• “4” - pulse.eco LoRaWAN based sensor. version 2.
• “20001” - pengy device, version 1.
• “20002” - URAD Monitor device.
• “20003” - AirThings platform device.
• “20004” - sensor.community crowdsourced device.

The Status of a sensor provides information about its operational state, having one of the following values:

• “REQUESTED” - a user requested this location with a device ID, but not sending data yet.
• “ACTIVE” - the sensor is up and running properly.
• “ACTIVE_UNCONFIRMED” - the sensor is up and running.
• “INACTIVE” - the sensor is registered but turned off and ignored.
• “NOT_CLAIMED” - the sensor is registered, but so far not bound to an owner.
• “NOT_CLAIMED_UNCONFIRMED” - the sensor is registered, but so far not bound to an owner nor confirmed by the community lead.
• “BANNED” - the sensor is manually removed from evidence in order to keep data sanity.

For example, if we see the following row in the ‘sensors_metadata.csv’ file

01cf1cec-bf2d-41b3-8cd5-e8bd720f01b4,3,"41.993498532663594,21.44513139126384",Madjir Maalo,Madjir Maalo,ACTIVE

It would mean that the sensor with id “01cf1cec-bf2d-41b3-8cd5-e8bd720f01b4” is a pulse.eco WiFi based sensor, version 2 located at the exact geographical point with latitude 41.993498532663594 and longitude 21.44513139126384, has “Madjir Maalo” as description and as a comment and is active – up and running properly.

To incorporate geographical data into an analysis, it is necessary to link the measurement data from the first file with the metadata in this file using the **SensorId** column. This join allows for the association of each measurement with its corresponding sensor’s geographical location, enabling spatial analyses and visualization of pollution levels across different areas. By combining these datasets, researchers can gain deeper insights into spatial trends and the geographic distribution of air quality parameters.

The data that is being collected, i.e. the data in the available dataset, is raw as it comes. No processing or cleaning methods have been applied to it (including handling missing values and outliers). In the following table we can see the general statistics of the measurements (Table 1).

**Table 1:** General statistics for the measured data.

Type	No. of values	Mean	Median	Min	Max	Std Dev	Skewness	Kurtosis	IQR
co	135,898	0.82	1.00	0.00	54.00	1.28	6.16	113.54	1.00
humidity	5,399,216	55.41	54.00	0.00	100.00	18.60	0.37	−0.24	25.00
no2	152,433	20.74	15.00	0.00	434.00	20.46	1.92	6.66	24.00
noise	5,278,480	59.16	44.00	0.00	215.00	42.57	1.38	1.20	48.00
o3	154,181	32.24	23	0	220	29.49	1.12	0.82	42
pm10	7,411,206	27.6	14	0	1999	47.72	10.16	226.09	22
pm25	7,343,330	16.68	8	0	999	28.53	9.99	220.74	14
pressure	2,875,054	981.77	984	247	1588	13.52	−2.29	35.9	10
temperature	5,478,687	17.45	17	-93	84	9.2	0.2	−0.5	14

From this table we can see that there are some values, especially for the atmospheric data, that are out of the expected normal range. This is due to the use of low-cost sensors, since the main purpose is to gather more data through crowd-sensing. This needs to be taken into consideration that when using the data for analysis, it should be properly preprocessed, especially when it comes to cleaning outliers.

**Fig. 1:**
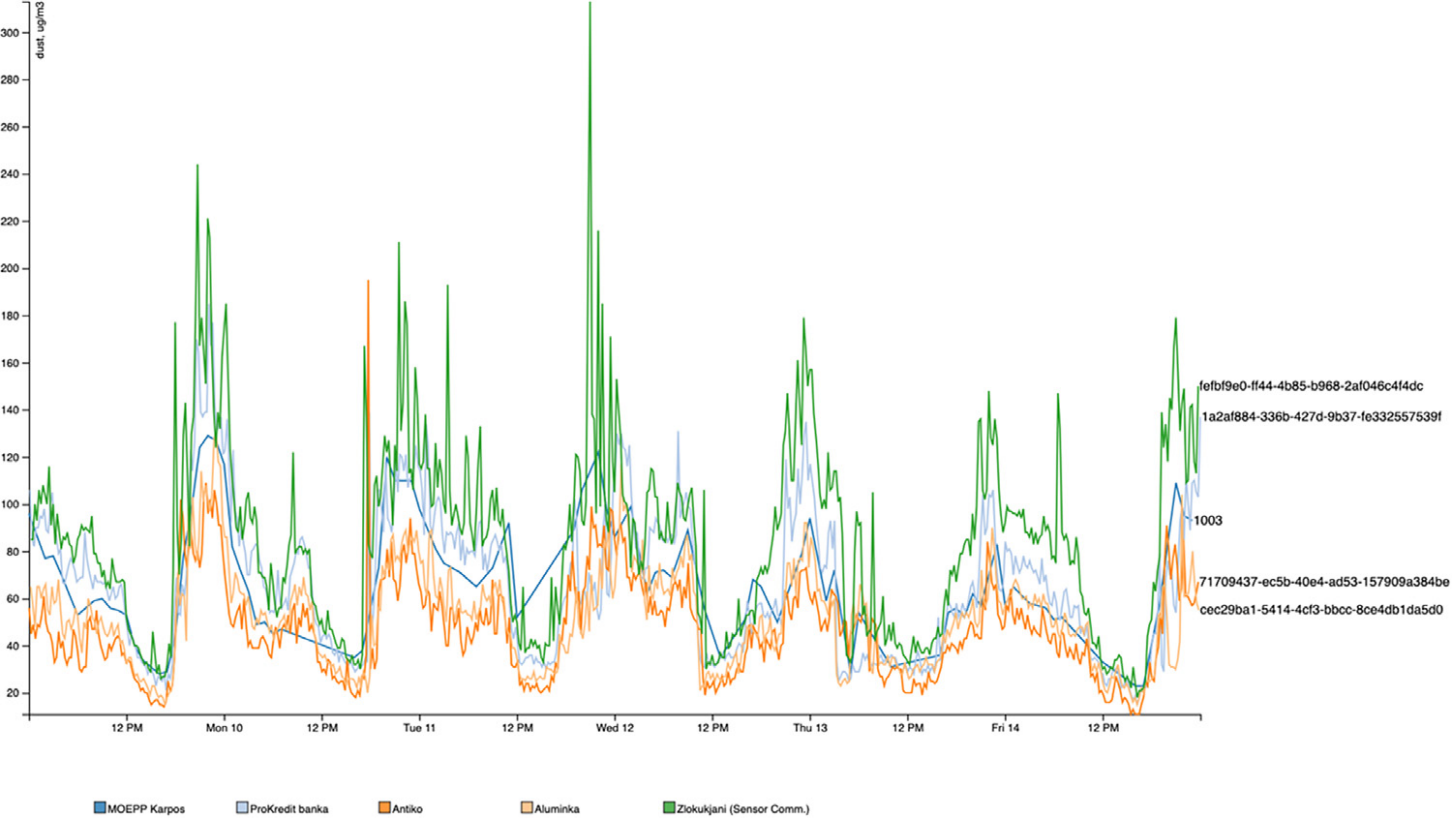
Pollution values of MOEPP Karposh station and surrounding sensors.

When it comes to the quality of the data, we have done some calibration comparations using the government owned gravimetric stations (MOEPP), which can be identified according to the type of the sensor as explained above – type “0”, as a base, and comparing surrounding low-cost sensors. For example, on Fig. 1 we can see a graph compering the MOEPP Karposh station with surrounding sensors: Zlokukjani – a nearby suburban area; ProKredit banka, which is right at the river, neighboring the urban Karposh area with Zlokukjani; Antiko and Alumnika – both are in the urban city area. It’s evident from the graph that the numbers are following the same trend, and that the devices pointing more to the suburban area are with higher, while the others with lower values.

We also did a Cointegration test using the MOEPP Centar government station as base and testing the two sensors closest to it – a community sensor in the center of the city, and the Majka Tereza 5 pulse.eco sensor. For both of them we got a *P*-value that is well below 0.05, which suggests that both of them are following a very similar long-term trend as the base sensor.

At the early years of the dataset, most of the data is from the urban city areas, since the sensors are mainly distributed there. However, as the network is expanded, more datapoints start to appear outside those areas as well. In the last year, the number of devices rose drastically both in the central urban areas and outside of them.

All devices started transmitting data once they were installed, meaning there is no mutual starting point in time for all locations. To note again that this is a crowsourced network, so both shorter and longer data outages, as well as sensors completely disappearing are to be expected by default.

**Fig. 2:**
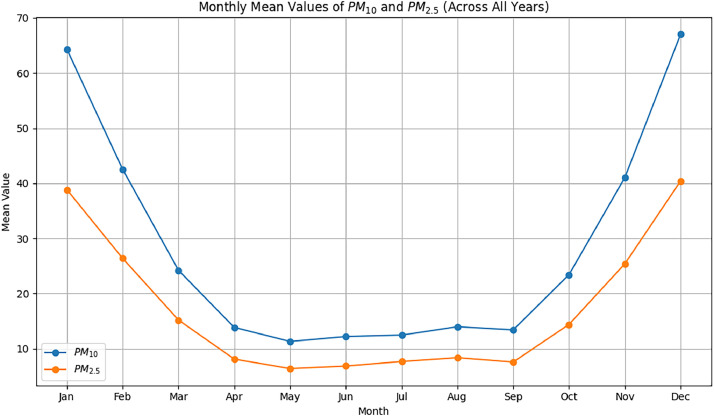
Monthly Mean Values of PM_10_ and PM_2.5_.

On Fig. 2 we can see a graph showing the monthly averages of PM_10_ and PM_2.5_ pollutants.

## 4 Experimental Design, Materials and Methods

Data collection is ongoing and automated, with sensor calibration comparative studies and checks performed periodically to ensure accuracy. The dataset covers a wide range of conditions, including seasonal variations, temperature fluctuations, and pollution peaks during traffic rush hours and industrial activities. This continuous monitoring system is vital for detecting pollution trends and informing policy interventions aimed at improving air quality.

**Fig. 3:**
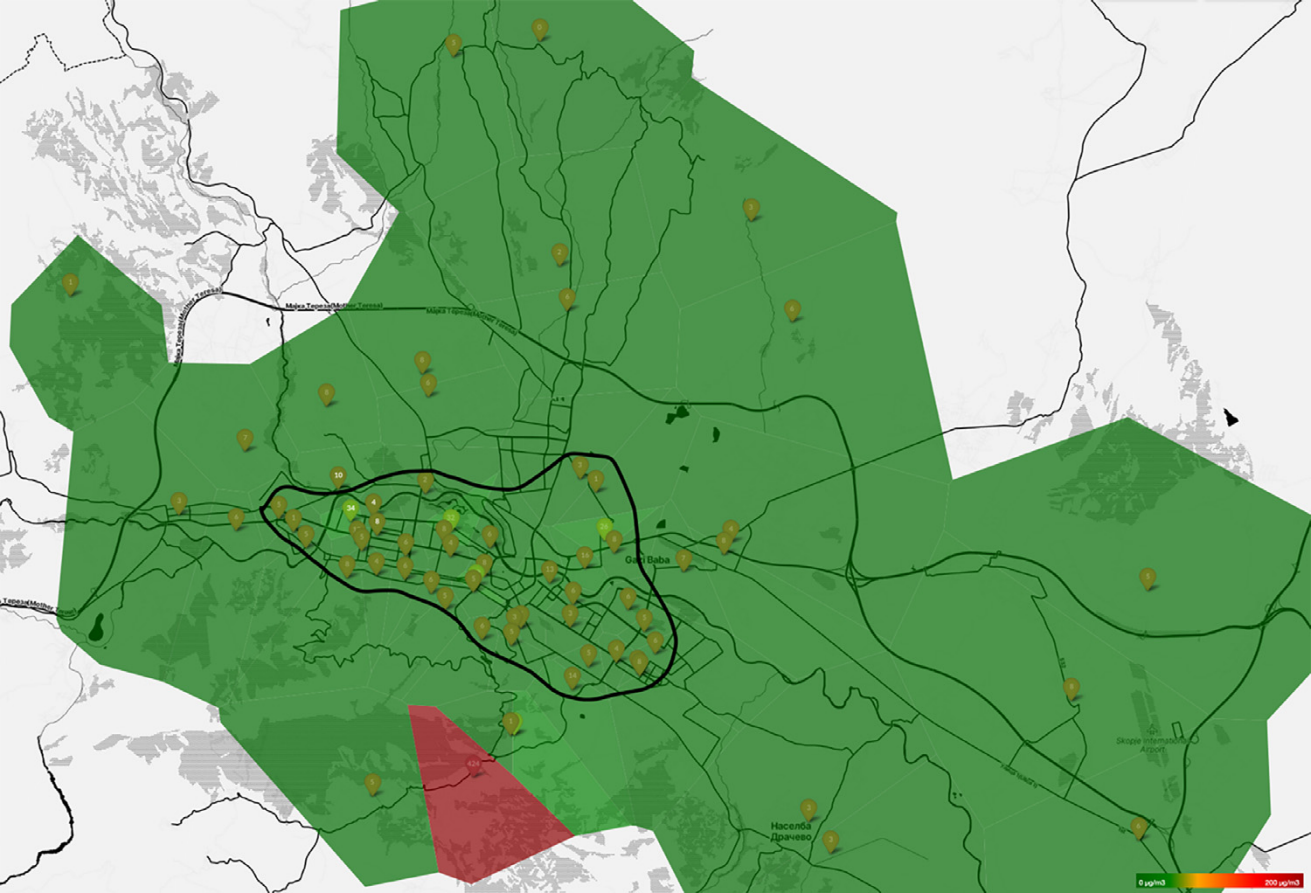
Map of the Skopje sensor network of pulse.eco.

The core of Pulse.eco’s capability lies in its crowd-sensing approach [10], which involves deploying a large number of low-cost sensors across various locations in a city. These sensors are placed on fixed installations on the balconies or in the yards of residences of people that have volunteered to be part of the network. They are always positioned between ground and fourth floor. On Fig. 3 we can see the distribution of the sensors throughout the city and its suburbs. The marked territory is the expanded-central city urban area.

From here we can see that most of the sensors are distributed in the outlined expanded-central city urban area, but the outskirts of the city are covered as well. Besides, even the central locations cover different conditions – almost 10 devices on the slope of a mountain, as well as some devices outside of the central heating area.

There are a few rules (guidelines) given to the people regarding the placement of the sensors:

• The device needs to be placed outside (balcony or yard), fixed to a wall or a post, and protected from direct sunlight and rain.
• The network coverage (WiFi or TTN LoRaWAN, depending on the type of the device) needs to be solid and reliable.
• The device needs to be placed away from active sources of contamination (anything that produces smoke, vibration or sound, e.g. ashtray, air conditioner, chimney, very close construction site etc.).
• The device needs to face the louder side of the building, so that the noise measurements are more realistic.
• The device must not be installed very high (more than 3 or 4 stories) because the air pollution measurement will not be realistic.

The sensors are factory pre-calibrated. No further calibration is done so that no unwanted tampering occurs. At end of life or when they are noticed to produce too many off values, sensors are discarded.

**Fig. 4:**
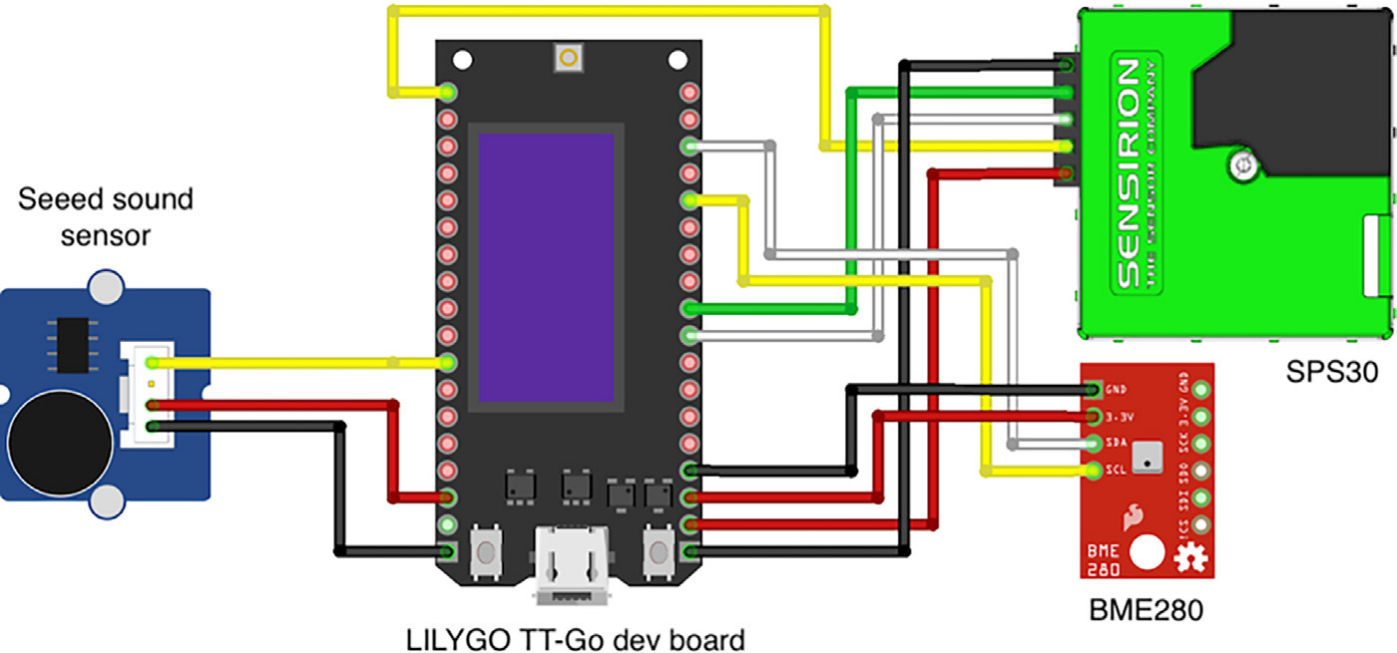
Wiring diagram of an ESP32 based pulse.eco sensor device.

Each sensor node can measure all the aforementioned types values through properly wired (Fig. 4) components described below.

• **ESP32 Microcontroller**: A powerful, low-power microcontroller with integrated WiFi and Bluetooth capabilities [11]. It acts as the central processing unit of the sensor node, collecting data from various sensors and managing communication with the backend. In our case, the LILYGO TTGO is used. It comes packed with ESP32, LoRa module, antenna connector, OLED mini screen, and multiple exposed GPIO ports as board pins. The integrated LoRa module enables long-range, low-power communication, making it ideal for wide-area urban monitoring, while WiFi provides a reliable option for data transmission in areas with strong local network availability. This dual-mode communication approach ensures a robust data delivery across diverse urban environments [12].
• **Particulate matter sensor** Each device is connected to a Sensirion SPS30 PM sensor [13]. The SPS30 is a compact PM_10_, PM_4_, PM_2.5_ and PM_1_ sensor that operates on laser scattering and is especially fit for this usage due to his long operating life and self cleaning capabilities.
• **Temperature, humidity and air pressure sensor**: A composite sensor module based on the Bosch BME280 is connected to provide data about the ambiental temperature, relative humidity and air pressure [14].
• **Noise sensor**: Last, for sensing the ambient noise, a Seeed Sound Sensor is packed in the device configuration as well. The sensor measures raw sound energy which is post-processed to resemble urban noise measure in dBAs [15].

## Limitations

As described above, one of the biggest limitations is the data quality, due to the use of low-cost sensors.

## Ethics Statement

The data collected is publicly available and does not involve human sub- jects. The Cleanbreathe project complies with ethical guidelines for data usage and environmental monitoring.

## CRediT Author Statement

**Slave Temkov:** Writing – Original draft, Formal analysis, Visualization, Writing – Review & Editing. **Pance Cavkovski:** Software, Writing – Review & Editing. **Petre Lameski:** Conceptualization, Writing – Review & Editing, Funding acquisition, Supervision. **Eftim Zdravevski:** Writing- Review & Editing, Resources, Validation. **Michael A. Herzog:** Conceptualization, Funding acquisition. **Vladimir Trajkovik:** Conceptualization, Writing – Review & Editing, Funding acquisition, Validation.

## Data Availability

Air pollution data: A dataset gathered through a crowd sensing platform (Original data) (https://repository.ukim.mk/).
